# Description of the unusual digestive tract of *Platax orbicularis* and the potential impact of *Tenacibaculum maritimum* infection

**DOI:** 10.7717/peerj.9966

**Published:** 2020-09-24

**Authors:** Maud Alix, Eric Gasset, Agnes Bardon-Albaret, Jean Noel, Nelly Pirot, Valérie Perez, Denis Coves, Denis Saulnier, Jehan-Hervé Lignot, Patricia N. Cucchi

**Affiliations:** 1 MARBEC, Univ Montpellier, CNRS, Ifremer, IRD, Montpellier, France; 2 Institute of Marine Research, Bergen, Norway; 3 Ifremer, UMR Ecosystèmes Insulaires Océaniens, UPF, ILM, IRD, Tahiti, French Polynesia; 4 BCM, Université de Montpellier, CNRS, INSERM, Montpellier, France; 5 IRCM, Université de Montpellier, ICM, INSERM, Montpellier, France

**Keywords:** Digestive system, Tenacibaculosis, Mucous, Osmoregulation, Absorption, Batfish, Teleost

## Abstract

**Background:**

Ephippidae fish are characterized by a discoid shape with a very small visceral cavity. Among them *Platax orbicularis* has a high economic potential due to its flesh quality and flesh to carcass ratio. Nonetheless, the development of its aquaculture is limited by high mortality rates, especially due to *Tenacibaculum maritimum* infection, occurring one to three weeks after the transfer of fishes from bio-secure land-based aquaculture system to the lagoon cages for growth. Among the lines of defense against this microbial infection, the gastrointestinal tract (GIT) is less studied. The knowledge about the morphofunctional anatomy of this organ in *P. orbicularis* is still scarce. Therefore, the aims of this study are to characterize the GIT in non-infected *P. orbicularis* juveniles to then investigate the impact of *T. maritimum* on this multifunctional organ.

**Methods:**

In the first place, the morpho-anatomy of the GIT in non-infected individuals was characterized using various histological techniques. Then, infected individuals, experimentally challenged by *T. maritimum* were analysed and compared to the previously established GIT reference.

**Results:**

The overlapped shape of the GIT of* P. orbicularis* is probably due to its constrained compaction in a narrow visceral cavity. Firstly, the GIT was divided into 10 sections, from the esophagus to the rectum. For each section, the structure of the walls was characterized, with a focus on mucus secretions and the presence of the Na^+^/K^+^ ATPase pump. An identification key allowing the characterization of the GIT sections using *in toto* histology is given. Secondly, individuals challenged with *T. maritimum* exhibited differences in mucus type and proportion and, modifications in the mucosal and muscle layers. These changes could induce an imbalance in the trade-off between the GIT functions which may be in favour of protection and immunity to the disadvantage of nutrition capacities.

## Introduction

This work brings new insights into the causes of orbicular batfish (*Platax orbicularis* (Forsskål 1775)) mortality by *Tenacibaculum maritimum* through a morpho-functional description of its digestive tract. The Gram-negative filamentous bacterium *T. maritimum* (formerly named *Flexibacter maritimus)* affects a large number of wild and cultured marine species (Avendaño Herrera, Toranzo & Magariños, 2006; Vilar et al., 2012; Faílde et al., 2013; Faílde et al., 2014; Pérez-Pascual et al., 2017; Gourzioti et al., 2018; Guardiola et al., 2019) and is one of the most limiting threats to fish farming due to its wide geographical distribution (Toranzo, Magariños & Romalde, 2005). It corresponds to the aetiological agent of the tenacibaculosis, an infection inducing gross lesions to the two first lines of defense against microbial infection: body surface and gills. Signs of disease include eroded mouth, skin ulcers, fin necrosis, and tail rot (Shephard, 1994; Ellis, 2001; Toranzo, Magariños & Romalde, 2005; Avendaño Herrera, Toranzo & Magariños, 2006; Gourzioti et al., 2018; Guardiola et al., 2019). Consequently, investigations on the tenacibaculosis mainly focused on the skin and gills (Chen, Henry-Ford & Groff, 1995; Handlinger, Soltani & Percival, 1997; Mitchell & Rodger, 2011; Småge et al., 2016), although few studies analysed the digestive system (e.g., Faílde et al., 2013; Faílde et al., 2014).

Indeed, the gastrointestinal tract (GIT), involved in digestion, nutrient assimilation, osmoregulation and immunity, acts as a barrier with the external environment (Wilson & Castro, 2010). Depending on the GIT region, the balance between the functions is not the same (e.g., jejunum and colon are mainly involved in nutrient and water absorption, respectively). However, the immune system is active throughout the GIT but more markedly in the ileum and colon with higher lymphoid tissue proportion (Gut Associated Lymphoid Tissue, e.g., Peyer’s patches in mammals) (Carroll, 2007; Ermund et al., 2013).

The mucus, a secretion of mucin, a large filamentous glycoprotein with a high level of glycosylation (Dekker et al., 2002; Bansil & Turner, 2018), has also a key role in the immune function as a selective protective film (Capaldo, Powell & Kalman, 2017). Its viscosity, which mainly depends on its carbohydrate nature, contributes to the trade-off between nutrient uptake, hydration or sealing, and the accumulation of antimicrobial peptides, antibodies, lymphocytes, commensal bacteria and luminal vesicles (Shephard, 1994; Gomez, Sunyer & Salinas, 2013; Birchenough et al., 2015; Bansil & Turner, 2018; Taherali, Varum & Basit, 2018). The mucus is secreted by mucous cells located in all epithelia of mucosal layers and integument. Depending on their location, lineage and secretion, the types of mucous cells differ. In the stomach, the neck mucous cells and surface mucous cells protect the mucosa of the gastric acid with a neutral mucin (Han & Oh, 2013). The rest of GIT contains goblet cells: mucous cell with a goblet shape (Taherali, Varum & Basit, 2018). The nature of the mucus secreted by these cells depends on the organ, species and environmental factors (Jonckheere et al., 2013).

*P. orbicularis*, a tropical species of high aquaculture value in French Polynesia, has become scarce in its natural habitat notably due to overfishing. Locally very appreciate for its meat taste and texture, *P. orbicularis* was a good candidate for launching a real fish farming sector in French Polynesia in 2011. The growing interest in this production (from 6.9 to 21.9 tons between 2011 and 2016 (DRMM, 2017; Andréfouët & Adjeroud, 2019)) is mainly due to the will to develop a sustainable aquaculture for this species of traditional consumption for Polynesian and Chinese (Gasset & Remoissenet, 2011). However, since 2017 the production dropped (10.4 tons in 2019, Pers. Com. Tahiti Fish Aquaculture) due to high mortality (up to 90%) shortly after the transfert of the juveniles from bio-secure land-based aquaculture system to the lagoon cages for growth. Among the causes, this mortality partly results from bacterial infections, including *T. maritimum* (Bardon-Albaret et al., 2015; Reverter et al., 2016) which was systematically identified using real-time PCR (Fringuelli et al., 2012; Bardon-Albaret et al., 2015) in fish exhibiting sign of disease (e.g., lack of apetite, white feces and whitish patches on the skin, Supplementary data 1). In addition, the analysis in microscopy of the lesions revealed long and rod-shaped filamentous bacteria typical of the tenacibaculosis infection (Saulnie D, Pers. Com., 2016). The GIT of *P. orbicularis* is compacted in a restricted space as occasionally observed in some flatfish species (Chanet et al., 2012; Kobelkowsky & Rojas-Ruiz, 2017). This characteristic is probably due to the disc-shaped morphology shared by the species from the *Ephippidae* family to which, *P. orbicularis,* belongs to Leu et al. (2018). Consequently, the GIT regions and therefore their functions are not always well characterized in the juveniles or adults.

In that context, a detailed characterization of *P. orbicularis* GIT is required to understand the regulation of its functions especially when juveniles are confronted for the first time to *T. maritimum*. Therefore, the main goals of this study are: (i) to characterize the morpho-anatomy of *P. orbicularis* GIT in non-infected juveniles and, (ii) to identify the impact of a challenge with *T. maritimum* on the functional structures of GIT at the organ, tissue and cellular level.

## Material and Methods

### Origin and management of animals

Animal experiments were carried out at the IFREMER experimental station located in Vairao (Tahiti, French Polynesia). For this study, 320 healthy *P. orbicularis* of 56 days post-hatching (dph) originating from the governemental VaiA hatchery (Vairao, Tahiti, French Polynesia) were used. In the absence of *adhoc* ethical committees in French Polynesia, *in vivo* experiments reported in the present study fulfill all the sections of deliberation no 2001–16 APF from the Assembly of French Polynesia insured in the Journal Officiel de Polynésie française on the 1st February 2001 dealing on domestic or wild animal welfare and followed animal care and ethic guidelines (Reilly, 2001; Barker et al., 2002; Kilkenny et al., 2010).

### Challenge with *T. maritimum*

#### Preparation of the *T. maritimum* inoculate

Pure bacterial culture of *T. maritimum* virulent strain TFA4 was incubated in nutrient medium (4 g L^−1^ peptone and 1g L^−1^ yeast extract Becton, Dickinson and Company, Sparks, MD in filtered and UV-treated sea water) for 48 h at 27 °C until reaching the stationary phase (i.e., interruption of bacterial division process). Bacterial concentration (CFU/mL) of the inoculate was evaluated by the plate-counting method on nutrient medium supplemented with 1.5% agar using appropriate dilution in sterile seawater.

#### Experimental design

Fish were maintained into twelve 150 L-tanks (six per experimental group with 25 to 26 individuals per tank). Two experimental groups of fish were applied: (i) non-infected and (ii) infected individuals corresponding to animals challenged by immersion in the culture medium without bacteria or with *T. maritimum* strain TFA4, respectively. The challenge occurred into 40 L-tanks containing 20 mL of either cultured *T. maritimum* strain TFA4 at final concentration of 5 × 10^4^ CFU/mL or culture medium without bacteria in which fishes were transferred and kept for 2 h. Then, the animals were removed from the 40 L-tanks, rinsed twice with UV-filtered sea water before returning to their respective 150 L-tanks. Animals were fasted 24 h prior the challenge, and refed from day 1 to 5 post-treatment, once a day.

For each experimental group, animals were either (i) monitored for post-treatment cumulated mortality (from day 1 to 5 post-treatment (pt), in %) or (ii) sampled for further analyses (see below). Mortality was used as an endpoint because reproducible challenge of *P. orbicularis* with a sublethal dose is not controlled, probably due to the high virulence of *T. maritimum* strain used. From the interruption of mortality events, 1 to 2 individuals exhibiting signs of disease (e.g., lack of apetite, white feces and whitish patches, supplementary data 1) were sampled in each tank. Fish were euthanized using an overdose of Benzocain (150 mg L^−1^ EtOH). This method of euthanasia, reproducible and safe to the operator, induces a depression of the central nervous system activity, rapid unconsciousness and death of *P. orbicularis*, without compromising further histological analyses.

#### Water quality

Fish tanks were supplied with filtered and UV-treated (300 mJ/cm^2^) sea water coming from the experimental site in the lagoon. Daily, one third of the 150 L-tank water was replaced with filtered and UV-treated sea water to maintain good water quality. Therefore, pH value were kept at 7.8 and N-NH4 maintained below 0.5 mg L^−1^. Tanks were supplied with air. Water temperature average was 28.0 ± 0.4 °C during the experiment.

### Histology and Tissue Micro-Array (TMA)

#### Sampling and dissection

**Figure 1:**
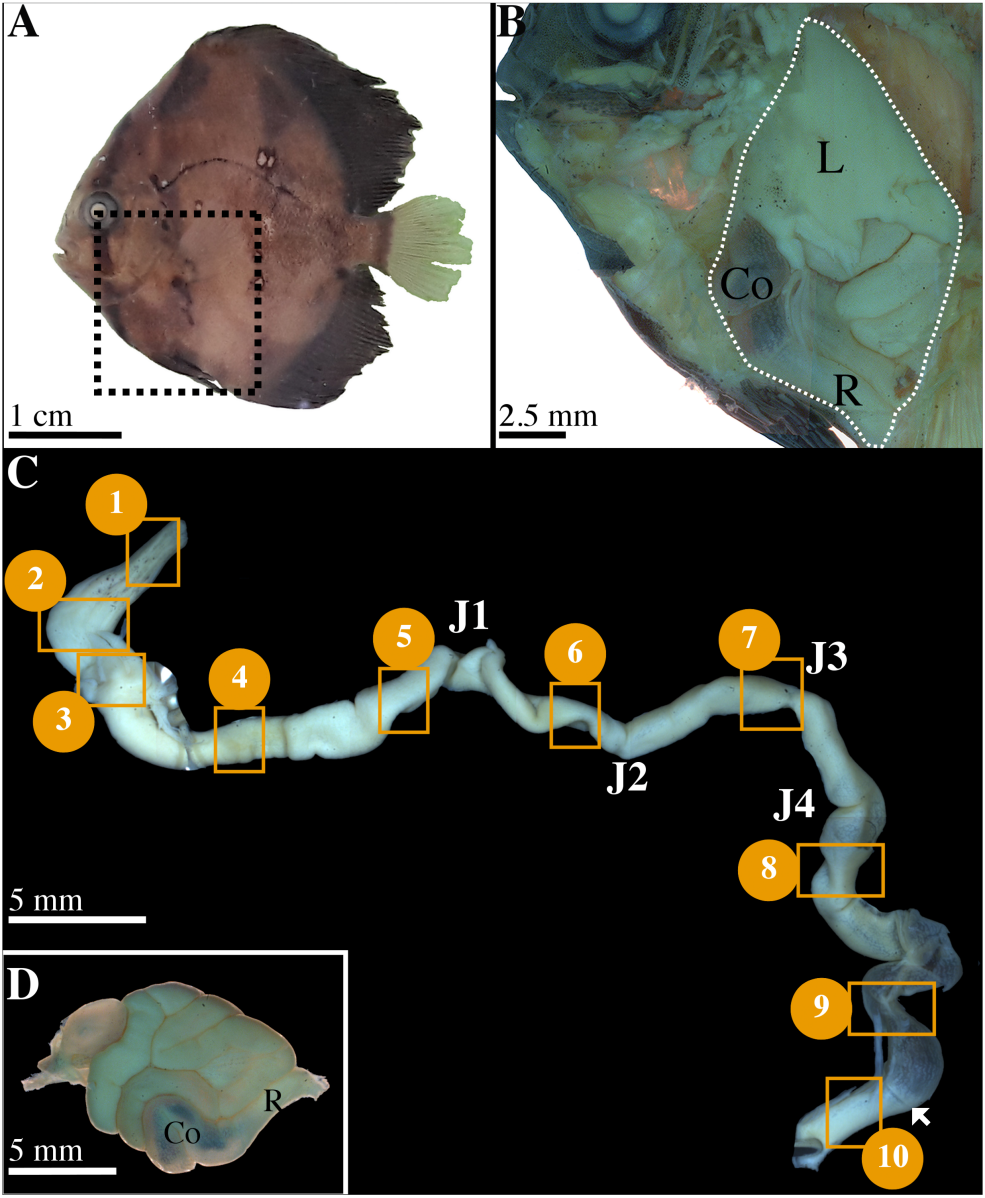
Characterization of gastrointestinal tract (GIT) sections of *P. orbicularis* and details of dissection. (A) 56 dph *P. orbicularis*. Dashed square represents the limits of the dissection, see Fig. 1B. (B) View of the GIT inside *P. orbicularis*. Dashed white line delimits GIT. (C) Unrolled GIT. Yellow squares represent sections of interest for this work. These sections have been numbered from 1 to 10. J1 to J4: junction 1 to 4. The white arrowhead shows the valve between the colon and the rectum. (D) Isolated GIT after dissection. Co, colon; L, liver; R, rectum.

**Figure 2:**
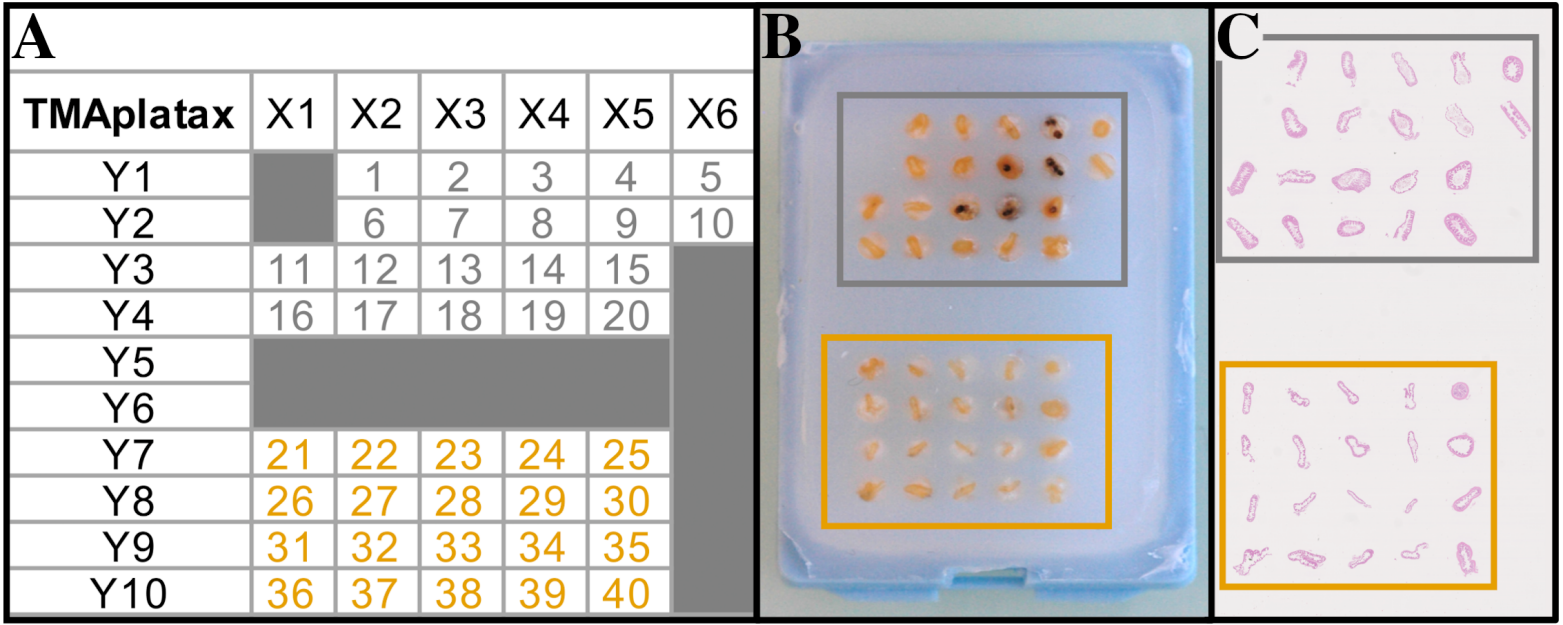
Tissue Micro Array design (TMA). (A) TMA plan. (B) TMA Recipient block. (C) Scanned slide of TMA after HES staining. Grey and orange squares/numbers correspond to non-infected and infected fish, respectively.

Fishes at 56 dph were fixed *in toto* in Davidson’s fixative for 48 h at 4 °C before being washed and conserved in 70% ethanol. Four non-infected and infected fishes were dissected to extract, unroll and image the GIT (Figs. 1A–1D) to distinguish the different parts using a stereoscopic microscope (Leica Wild M420, magnifications ×5.8 to ×16). Images were captured using a Leica DC 300F digital camera associated to the FW 4000I software (Leica Microsystems, https://www.leica-microsystems.com/). According to the literature, the morphology of digestive convolutions and junctions observed in light and scanning electron microscopy (SEM), the GIT was divided into 10 different sections (Figs. 1C, 2). The sections 1 to 5 were identified prior the junction (J) 1 (Fig. 1C). The sections 6 and 7 were located between J1/J2 and J2/J3, repectively (Fig. 1C). Finally, the position of the sections 8 to 10 was determined after the last junction, J4 (Fig. 1C). In total, 80 samples were obtained (10 sections ×8 fish).

#### Paraffin embedding and Tissue Micro-Array (TMA)

Isolated GIT sections were progressively dehydrated in ascending series of alcohol, orientated on transversal section and paraffin embedded. The TMA method used consists in coring automatically the GIT sections in each donor block using a tissue arrayer (TMA Master II^™^, 3DHistech LTD) and inserting them in a recipient block (Figs. 2A–2B). From the 80 donor blocks, two recipient blocks of TMA were designed containing 40 samples each (5 sections per animal, from sections 1 to 5 and sections 6 to 10 for block 1 and 2, respectively) (Fig. 2A). Then, three-micrometer sections were obtained from the recipient blocks using a rotary microtome (Microm HM 340E, Thermo Fisher Scientific) (Fig. 2C). Slides were conserved at 4 °C until treatment.

#### Classical staining

Hematoxylin –Eosin –Saffron (HES) staining was performed using an automated autostainer (Microm HMS740, Thermo Fisher Scientific). Slides were progressively rehydrated and tissues were stained with Mayer hematoxylin 1.5X (5 min), alcohol eosin 0.5% (7 min) and saffron (6 min). Slides were dehydrated and mounted in Mounting medium Pertex^®^Histolab. In addition to HES staining, a classical Masson’s trichrome (TC) staining protocol was used to highlight collagen fibers (Martoja & Martoja-Pierson, 1967).

The identification of the mucous cells was done according to the original PAS-AB pH2.5 protocol (Yamabayashi, 1987) using an automated autostainer (Myreva SS-30, Myr, Microm Microtech France). This common histological technique allow distinguishing 3 types of secretion according to their coloration: neutral, acidic or mixed mucus. Moreover, it allows a fast and inexpensive initial characterization of mucus and is still widely used (Bates et al., 2006; Zhang et al., 2018; Kalhoro et al., 2019; Yang et al., 2019).

#### NKA immunolabeling

NKA-ATPase localization was determined using immunolabeling according to the protocol adapted from Ituarte et al. (2016). Before to block non-specific binding, the sections were placed in a citrate-buffered solution, pH 6, heated up in the microwave (10 min) to expose the epitopes. Afterwards, they were incubated in a humidity chamber at 4 °C overnight with the rabbit anti-Na^+^/K^+^-ATPase H300 primary antibody (Santa Cruz Bio-technology) diluted in phosphate buffered saline containing Régilait^®^ (PBS-R 0.5%) at 4 µg/mL. To remove the excess of antibody, the sections were washed in PBS before being incubated with the secondary antibody (Alexa Fluor^®^488 donkey anti-rabbit, Invitrogen) at 10 µg/mL in PBS-R 0.5% for 1 h at room temperature. Sections were mounted in an anti-bleaching medium (ImmunoHistoMount, Aqueous-based Media, Santa Cruz Bio-Technology). Negative control was made with the secondary antibody alone and positive control was made by Western Blot with specific NKA antigen (Ituarte et al., 2016).

### Morphometry and image analyses

#### Light microscopy

Histological sections for classical stainings were photographed using a Leica DM 2000 LED microscope equipped with a camera Leica MC170 HD (Leica Microsystems, https://www.leica-microsystems.com/). Three micrographs per fish were taken at the x20 magnification and used for measurements in the relevant GIT sections using the Image J software (v. 1.52, http://rsbweb.nih.gov/ij/). Epithelial thickness was calculated using enterocyte length from the basal membrane of the cells to the apical border of the microvilli. Total muscle thickness was calculated adding the measurements of longitudinal and circular muscle layers. Epithelial and muscle thickness data resulted from the average of 30 measures/fish (10 measures/picture). Four fishes were used for each group. Mucous cells density (number of mucous cells per µm of epithelium) and the proportion of acid and neutral mucous cells (in %) were calculated in the relevant sections.

#### Scanning Electron Microscopy (SEM)

GIT sections opened longitudinally, were dehydrated through a graded ethanol series, then bathed in hexamethydisilazane (1 min, twice) and air-dried. They were attached to stubs using adhesive carbon tape. The samples were coated with gold for 180 s and examined with a FEI Quantum 200 ESEM using a conventional mode (high vacuum, 10 KV) and Thornley-Everhart secondary electron detector.

#### Confocal microscopy

Immunolabeled sections were examined and imaged at the x25 Fluostar 0.75 IMM objective with 1.5 zoom using a Leica SP5 confocal microscope together with the Leica LAS X software (Leica Microsystems) in the Montpellier RIO Imaging Facility. Background was adjusted with the negative control. All the 1024 by 1024 pixels 8-bit pictures were taken under the same settings in the 2 TMA slides: laser line 488 at 14% of intensity, PMT Gain 872%, Offset 0%, emission bandwidth 510 to 533 nm, pinhole aperture 94.4 µm with airy 1 and 6 frames average picture.

### Statistical analysis

Statistical analyses were performed using the software “R” (version 3.6.1). The assumption of normality and homogeneity of variances were tested for all measured variables. When data did not respect the assumption of normality, a non-parametric Wilcoxon test was used to determine a significant difference between infected and non-infected groups. The minimum level of significance was set at *p* < 0.05. Average measurements for each group are presented in barplot ± standard deviation.

## Results

To broaden our comparison, we employed terms commonly used in many vertebrates: duodenum, jejunum, ileum and colon corresponding to the anterior, mid and posterior intestine. Ileum could be considered as the most distal part of mid-intestine. The presence of villi is very rare in fish thereby the terms primary and secondary folds will be used (Wilson & Castro, 2010).

### Functional anatomy of the GIT in non-infected *P. orbicularis*

The GIT of *P. orbicularis* has the particularity of being curled up on itself in a restricted visceral cavity below the animal’s head (Figs. 1A, 1B) inducing many convolutions in the GIT. The direction of these convolutions and the position of the segments in the visceral cavity varies from one individual to another (Fig. 1C). However, in the observed individuals, they consistently appear with also the presence of at least 4 intestinal junctions (J1 to J4). These junctions served as benchmarks to determine the segments to be studied (Fig. 1C). The relative intestinal length (RIL = intestinal length/total body length) or intestinal coefficient (IC) is 1.47 ± 0.14 (mean ± SD).

**Figure 3:**
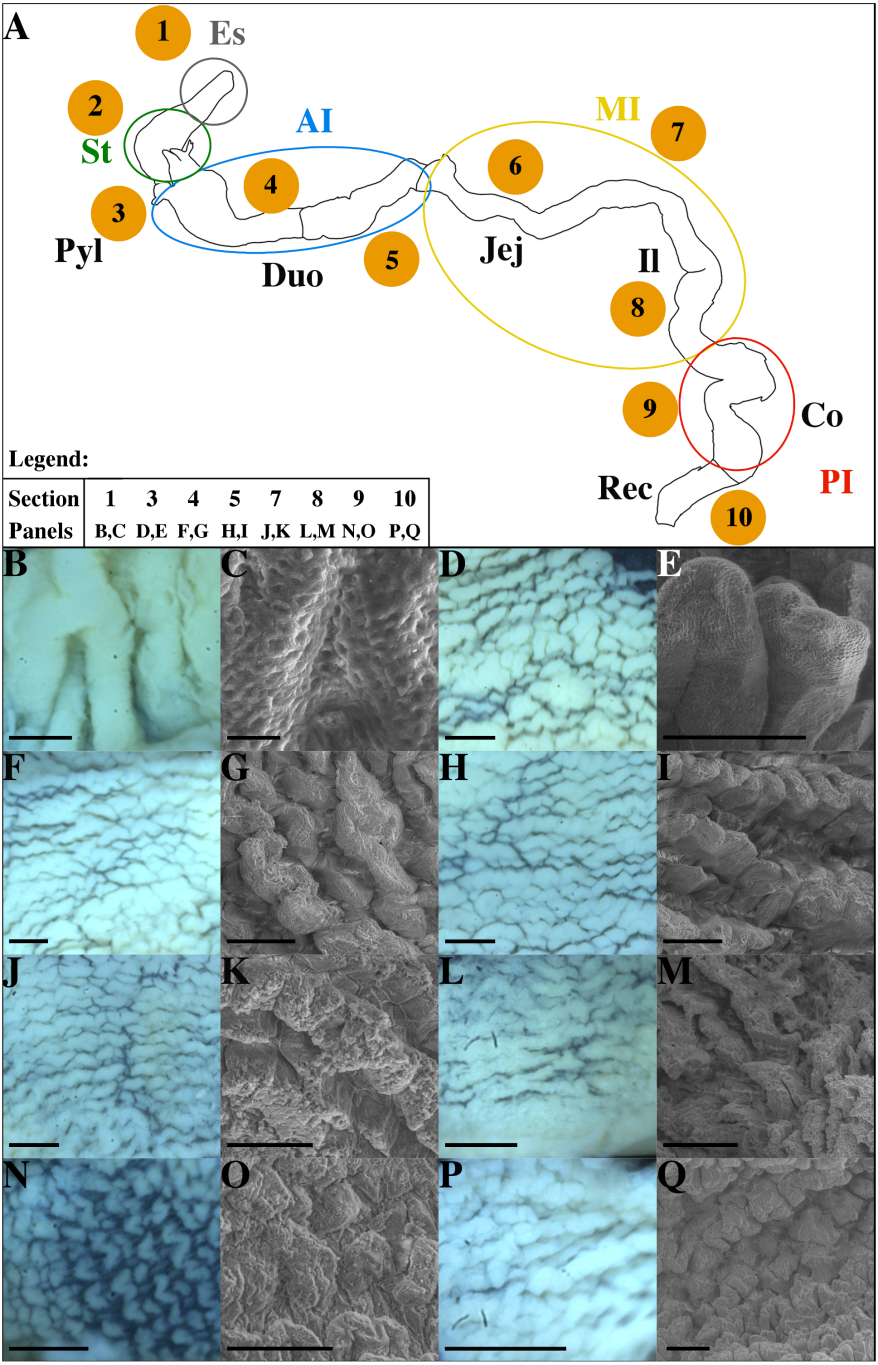
Schematic representation of the GIT in non-infected *P. orbicularis* (A) and microscopic characterization of sections studied (B–Q). (A) Numbers 1–10 correspond to sections defined in Fig. 1. Colored circles delimit the functional regions of the GIT. (B–Q) Light and scanning electron microscopy (SEM) pictures of the GIT sections. (B, D, F, H, J, L, N, P) Light microscopy which represent the gut folds, scale bars = 500 µm. (C, E, G, I, K, M, O, Q) SEM pictures, scale bars = 200 µm except for C where the scale bar = 10 µm. (B, C) Section 1. (D, E) Section 3. (F, G) Section 4. (H, I) Section 5. (J, K) Section 7. (L, M) Section 8. (N, O) Section 9. (P, Q) Section 10. AI, Anterior Intestine; Co, Colon; Duo, Duodenum; Es, Esophagus; Il, Ileum; Jej, Jejunum; MI, Medium Intestine; PI, Posterior Intestine; Pyl, pylore; St, stomach; Rec, Rectum.

When the GIT is unwound, few mesenteric cords and a diffuse pancreas are observed. The short esophagus (2.5 mm) is one mm wide and extends from the end of the pharyngeal cavity to the stomach (Figs. 1C, 3A, sections 1 and 2). It has scattered pigmented spots on its opaque and crenellated wall (Fig. 1C, sections 1). The stomach is J-shaped, wider (4 mm) and flat, with an opaque wall, also crenellated, slightly pigmented on the front part (Figs. 1C, 3A, sections 2). It ends up in the intestine at the level of the pylorus (Figs. 1C, 3A, sections 3).

A large liver covers on both sides the esophagus, stomach and pylorus (Fig. 1B). The gall bladder is located on the top of the liver, on the right side of the fish and the bile duct ends at the level of the pylorus.

Following the pylorus, the midgut or intestine begins and presents 4 pyloric caeca opening into its lumen. The intestinal length varies according to the individual (40 to 45 mm), its width is from 2 to 3 mm. The anterior intestine extending from the pylorus to the J1 (Fig. 1C), is presumed to correspond to the duodenum and is divided into the proximal (Figs. 1C, 3A, section 4) and distal parts (Figs. 1C, 3A, section 5). The mid-intestine is divided into the proximal (Fig. 1C, between J1 and J2, Fig. 3A, section 6), median (Fig. 1C at J3, Fig. 3A, section 7) and distal parts (Fig. 1C after J4, Fig. 3A, section 8). It is assumed that the first two parts correspond to the jejunum (Figs. 1C, 3A, sections 6 and 7), and the last one to the ileum (Figs. 1C, 3A, section 8). The posterior intestine or colon (10 to 15 mm) is easily identifiable due to its transparent wall (Figs. 1C, 3A, section 9). On the contrary, the rectum is characterized by a very dense and opaque wall (Figs. 1C, 3A, section 10). The rectum ends with the anus aperture between the two pelvic fins (Fig. 1B).

### Structure of digestive organs

**Figure 4:**
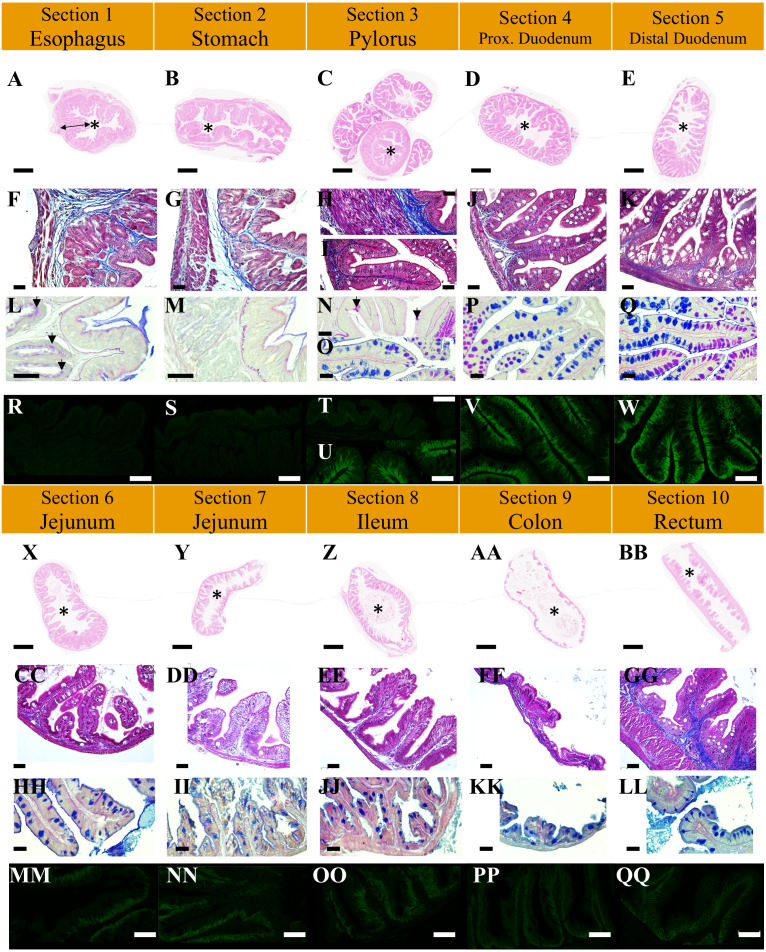
Structure of intestinal transversal sections in non-infected *P. orbicularis* in light microscopy (A to Q, X to LL) and confocal microscopy (R to W, MM to QQ). (A to E, X to BB) Tube structure, HES stain, scale bars = 500 µm. F to K, CC to GG: Wall tube structure, TC stain, scale bars = 25 µm. L to K, HH to LL: Mucous cell type, PAS-AB2.5 stain, scale bars = 25 µm. R to W, MM to QQ: Ionocyte, IF NKA, scale bars = 30 µm. A, F, L, R: Section 1, black double-side arrow indicates an example of primary fold, black arrow heads show mucous cells; B, G, M, S: Section 2; C: Section 3. H, N, T : Section 3 pylorus, black arrow heads show mucous goblet cells; I, O, U: Section 3 caeca; D, J, P, V: Section 4; E, K, Q, W: Section 5; X, CC, HH, MM: Section 6; Y, DD, II, NN: Section 7; Z, EE, JJ, OO: Section 8; AA, FF, KK, PP: Section 9; BB, GG, LL, QQ: Section 10. Asterisks (*) indicate the lumen of the section.

The esophagus and stomach (Fig. 3B) have large primary folds (PF) from 200 to 250 µm wide. The length of these PF can extend over the entire length of the organ. They often have forked branches. The PF height can reach 800 µm (Fig. 4A, black double-side arrow). On these folds, there are pits of 2 to 3 µm of external circumference and spaced every 2 to 4 micrometers (Fig. 3C) but no secondary folds (SF). The esophagus lumen has a circumference of 200–250 µm according to individuals (Fig. 4A, asterisk). The flattened J-shaped stomach (Fig. 1C) has an oval lumen of 100–150 µm by 2.5 to 3 mm (Fig. 4B). The intestine (Figs. 3D to 3M) has finer (20–30 µm), lower (70 to 250 µm) and shorter (100 to 700 µm) wave-shaped PF presenting SF (Figs. 3E and 4 Caeca in sections 3 and 4 to 10). The duodenum and jejunum (Figs. 3D to 3K) have similar PF (25–30 µm wide, 200–500 µm long) but slightly higher towards the pylorus (250 µm) with lower heights in the jejunum (200 µm). The duodenum has an oval cross-section with a lumen of 20–25 µm wide by 50–55 µm long and bounded SF (Figs. 4D, 4E). However, there seems to be a rather polar distribution difference in the most distal portion. This difference in distribution for the same cross-section is found in the jejunum, ileum and colon for some individuals but this is not systematic. The jejunum (Figs. 2C, 4X, 4Y) is flatter with a slightly larger lumen (10–25 µm wide by 75–80 µm long) than the duodenum due to smaller PF. The ileum (Figs. 3L, 3M) has long (up to 500 µm), thin, straight and flattened PF (70 µm) similar in size than those in the jejunum (Fig. 4Z). However, the lumen of the ileum is wider (10 to 45 µm) (Fig. 2C). The colon has a wide lumen (Figs. 2C, 4AA) varying according to the area and clearly visible and scattered wave-shaped PF (Fig. 3N). These PF (Fig. 3O) are relatively flat (50–200 µm) and short (70–450 µm). These SF are very small, flat and scattered (Figs. 3N and 3O). Finally, the PF observed in the rectum are thick, tight (Fig. 3P) and their heights are similar to those of the duodenum but difficult to evaluate using SEM (Fig. 3Q). The size of the lumen is variable (Figs. 2C and 4BB).

### Functional histology

**Figure 5:**
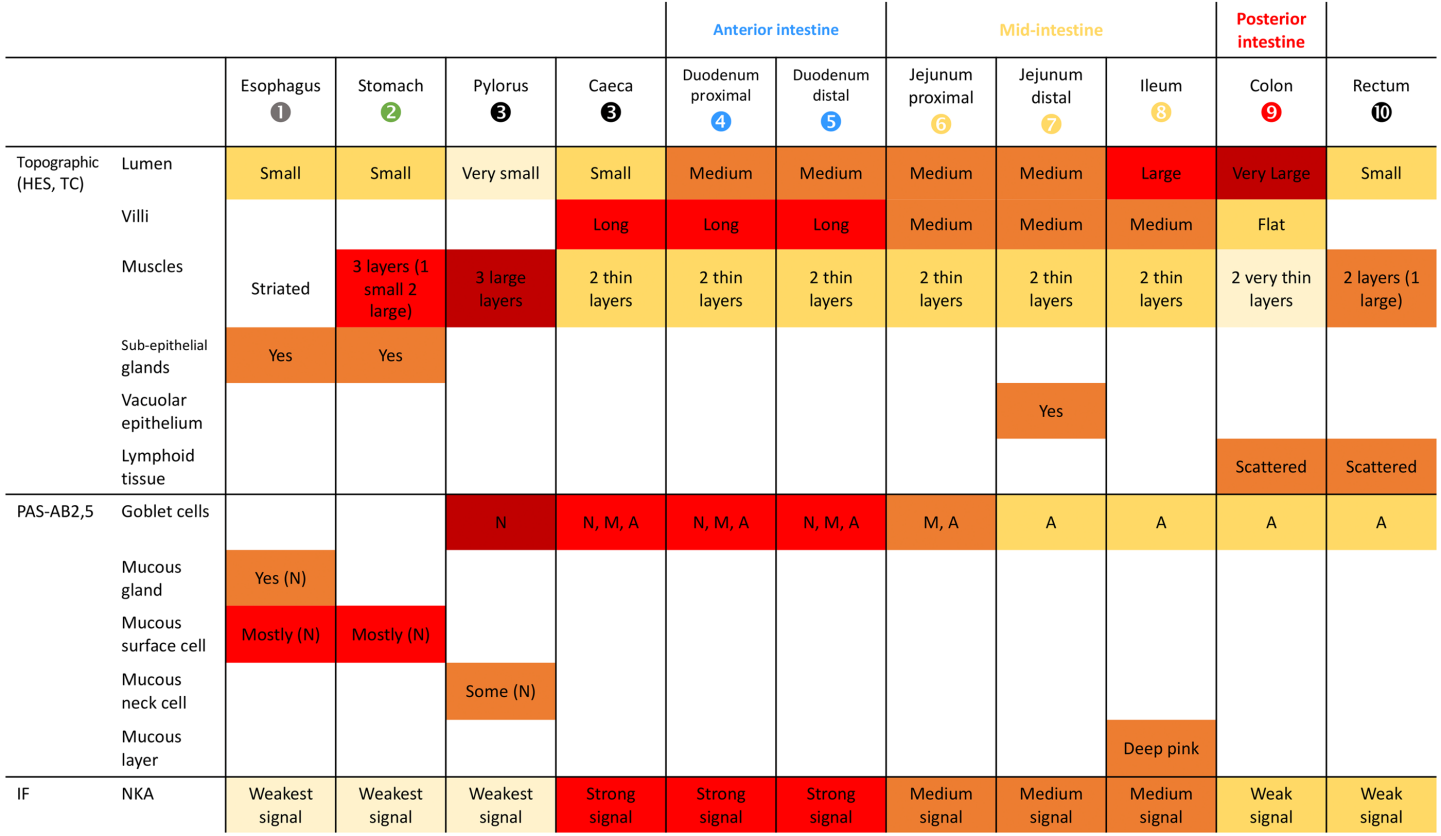
Identification key of the GIT of *P. orbicularis* (56 dph). The empty fields indicate the absence of the element. The goblet cells are defined by the letters A, M and N for the presence of acid, mixed or neutral mucus respectively.

Histological analyses with specific stainings (PAS-AB2.5, NKA-IF) provide topological identification keys (Fig. 5) characterizing the different parts of the GIT and their functionality (Fig. 4). Muscle organization allows the distinction of the esophagus and the stomach due to the presence of striated muscles (Fig. 4F) and of three layers of muscle (Fig. 4G), respectively. Although a third layer of muscle is observed in the pylorus (Fig. 4C) which is much thicker than in the stomach (Fig. 4H). Two thin layers of smooth muscle are observed in the intestine while the rectum is characterized by a thick layer of internal circular muscles (average of 118.9 µm, Fig. 4GG). Total muscle layer thickness decreases from the duodenum to the colon (36.7 to 29.9 µm) with a minimum in the ileum section (25.8 µm) and considerably increases in the rectum (167.2 µm). A valve between the colon and the rectum was also noted (Fig. 1C, arrow head). Moreover, the epithelium thickness progressively decreases throughout the GIT from the duodenum (60.2 µm) to the rectum section (35 µm).

Subepithelial tubular glands are present in the mucosa of the esophagus and stomach, but only the esophagus possesses mucous glands stained by the action of periodic acid and Schiff’s reagent (Figs. 4L, 4M). The majority of epithelial cells observed correspond to the surface mucous cells in stomach and esophagus (Figs. 4L, 4M). The presence of NKA is almost undetectable in these two sections (Figs. 4R, 4S, Data S2).

Mucous neck and surface mucous cells are observed in the pylorus section without sub-epithelial glands (Fig. 4N, arrow heads). The pyloric caeca and duodenum section are characterized by a strong presence of NKA along the baso-lateral membrane of the enterocytes (Figs. 4U, 4V and 4W). There is also a high density of polarized mucous goblet cells (Figs. 4O, 4P and 4Q). A neutral staining of the mucus close to the lumen at the apex of the folds is observed, with a mixed labelling in the middle parts and an acid staining at the base of the folds.

The remaining distal part of the GIT is characterized by a lower density of acid mucous goblet cells (the unique type found in the section) and a less intense NKA labelling. The distal part of the jejunum (section 7) is characterized by with numerous vacuoles at the enterocyte apex (Fig. 4DD and II). A dense staining of the epithelial layer and underlying connective tissue with Schiff’s reagent is noted in the ileum (Fig. 4JJ).

### Impact of *T. maritimum* on the GIT (6 days reaction)

**Figure 6:**
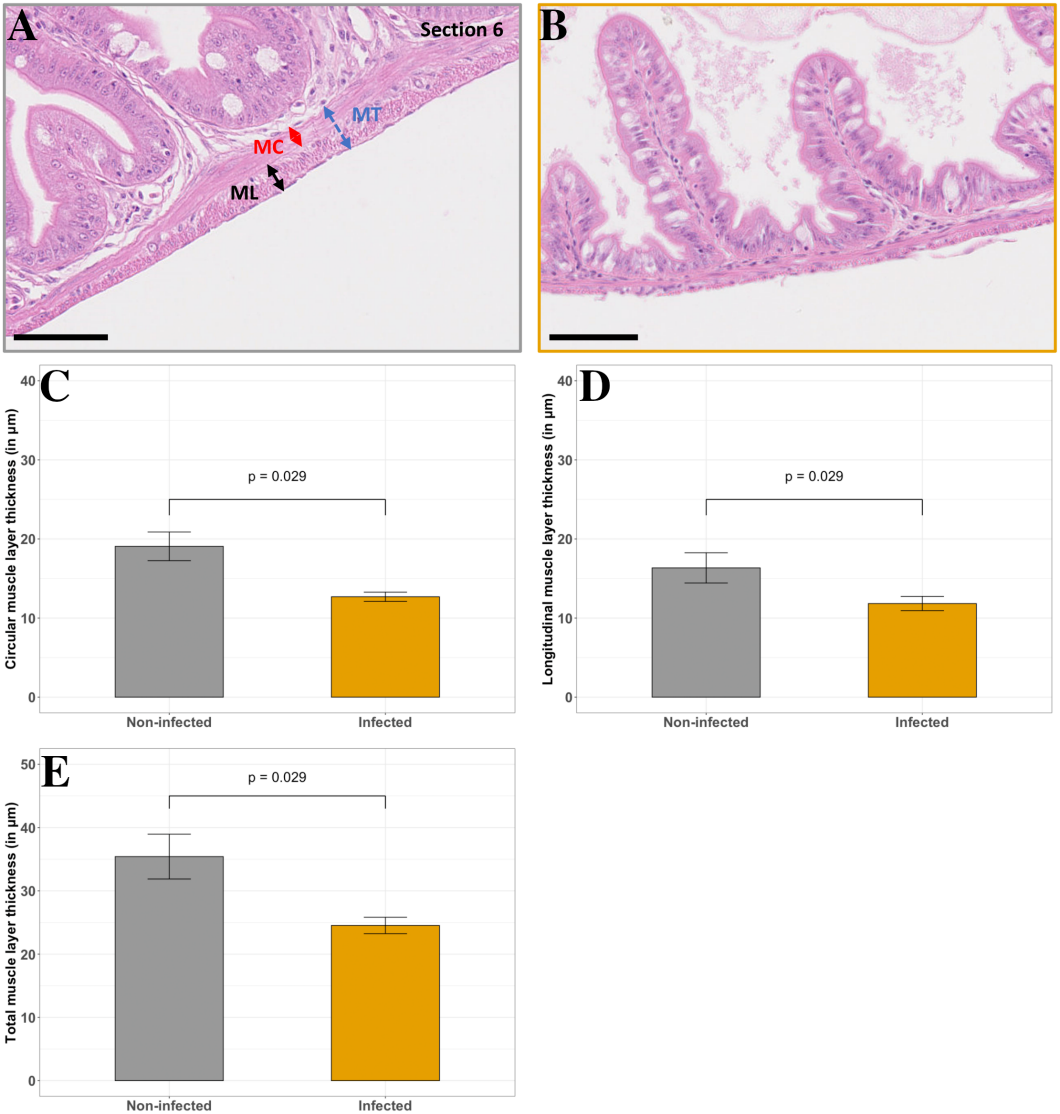
Comparison of non-infected and infected *P. orbicularis* muscle layers in the proximal jejunum (section 6). (A) Muscle layers in light microscopy in non-infected fish, (B) Muscle layers in light microscopy in infected fish. MC: circular muscle, ML: longitudinal muscle, MT: total muscle layer. Scale bars = 50 µm. Circular (C), longitudinal (D) and total (E) muscle layer thickness (in µm). Grey and orange bar plots correspond to the average of muscle layer thickness measurements for non-infected and infected fish, respectively. Measurements have been assessed on slides stained with HES as shown in A and B. Results of Wilcoxon test (*p*-value) is indicated for each comparison (*α* = 0.05, *n* = 4 per treatment).

No mortality was observed in non-infected fish (Table 1). In contrast, cumulated mortality increased from day (D) 0 to D5 pt for infected individuals, peaking at D2 pt (Table 1). In those fish, specific sections and structures of the GIT are affected. First, the thickness of the muscle layer are not affected by tenacibaculosis in most of the GIT sections except for the proximal part of the jejunum (section 6). Infected individuals possess significantly thinner muscle layers in section 6 (circular and longitudinal muscles) than non-infected fish (Fig. 6; *p* < 0.05, Wilcoxon test). In total, muscle thickness decreases by 31%, from 28% to 33% for longitudinal and circular muscle layer in jejunum, respectively. Moreover, inter-individual variability seems to be more important in non-infected individuals (Fig. 6).

**Table 1:** Cumulative daily mortality rates (in % ± SD) in non-infected and infected *P. orbicularis* from day 0 post-treatment (pt) to 5 days pt.

Days post-treatment	Non-infected	Infected
Day 0	0.0 ± 0.0	0.0 ± 0.0
Day 1	0.0 ± 0.0	1.3 ± 2.3
Day 2	0.0 ± 0.0	33.3 ± 9.2
Day 3	0.0 ± 0.0	53.3 ± 20.5
Day 4	0.0 ± 0.0	54.7 ± 22.5
Day 5	0.0 ± 0.0	54.7 ± 22.5

#### Impact on the mucous layer

**Figure 7:**
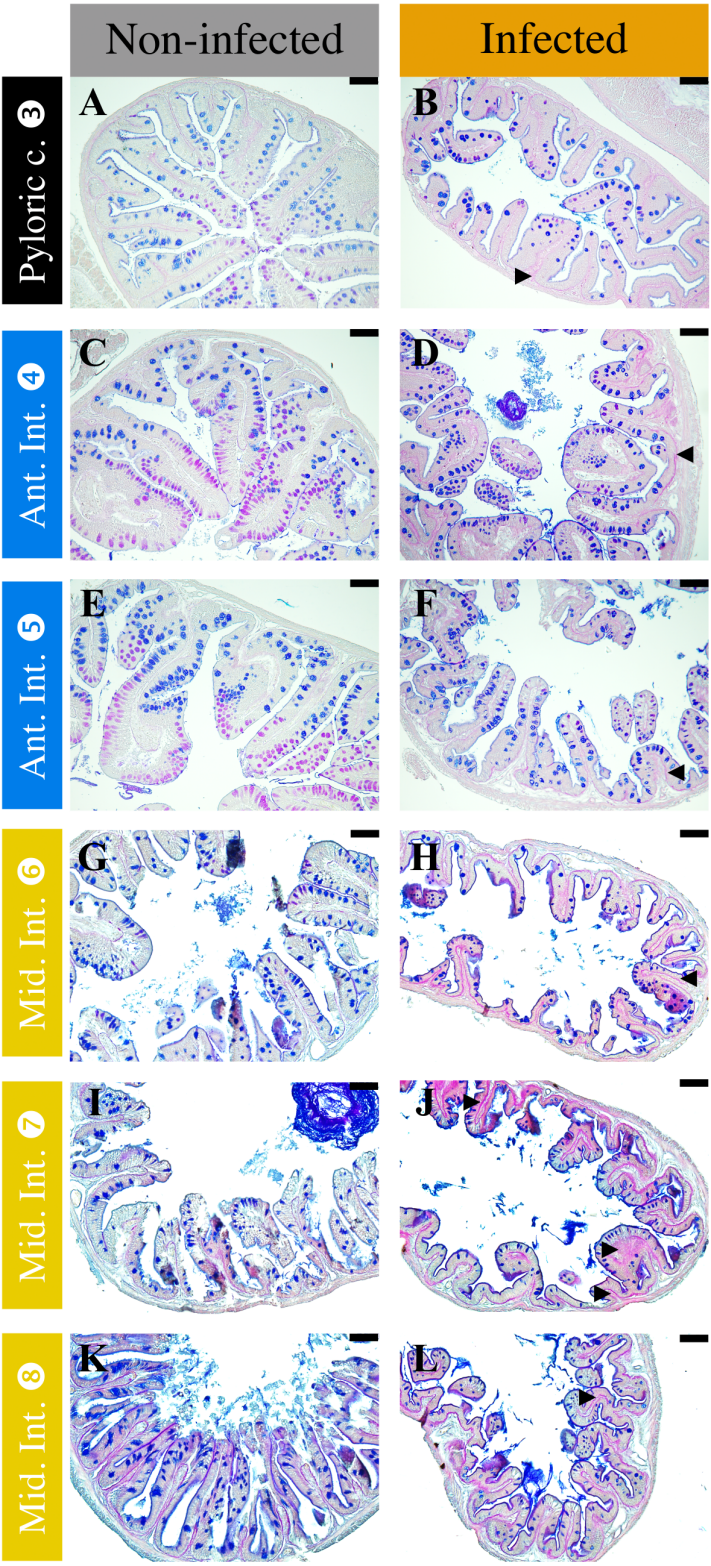
Comparison of non-infected (A, C, E, G, I, K) and infected (B, D, F, H, J, L) fish mucous cells and mucus composition in different sections of *P. orbicularis* GIT stained with PAS-AB 2.5. Acidic mucus is stained in blue and neutral mucus in magenta. Black arrow heads show differential staining in the infected fish relative to the not infected ones. Scale bars = 25 µm.

**Figure 8:**
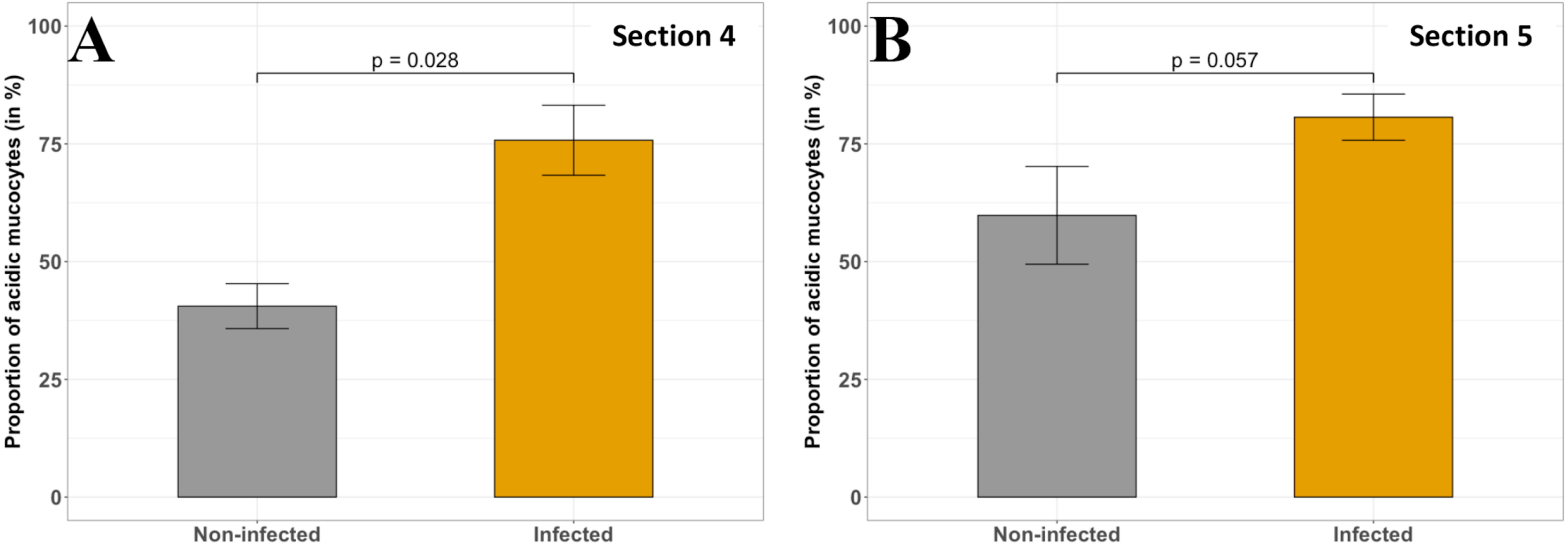
Comparison of the acid mucous cells proportion (in %) in non-infected and infected fish in different sections of *P. orbicularis* GIT. (A) section 4, (B) section 5. Measurements have been assessed on slides stained with PAS-AB 2.5. Results of Wilcoxon test (*p*-value) is indicated for each comparison (*α* = 0.05, *n* = 4 per treatment).

**Figure 9:**
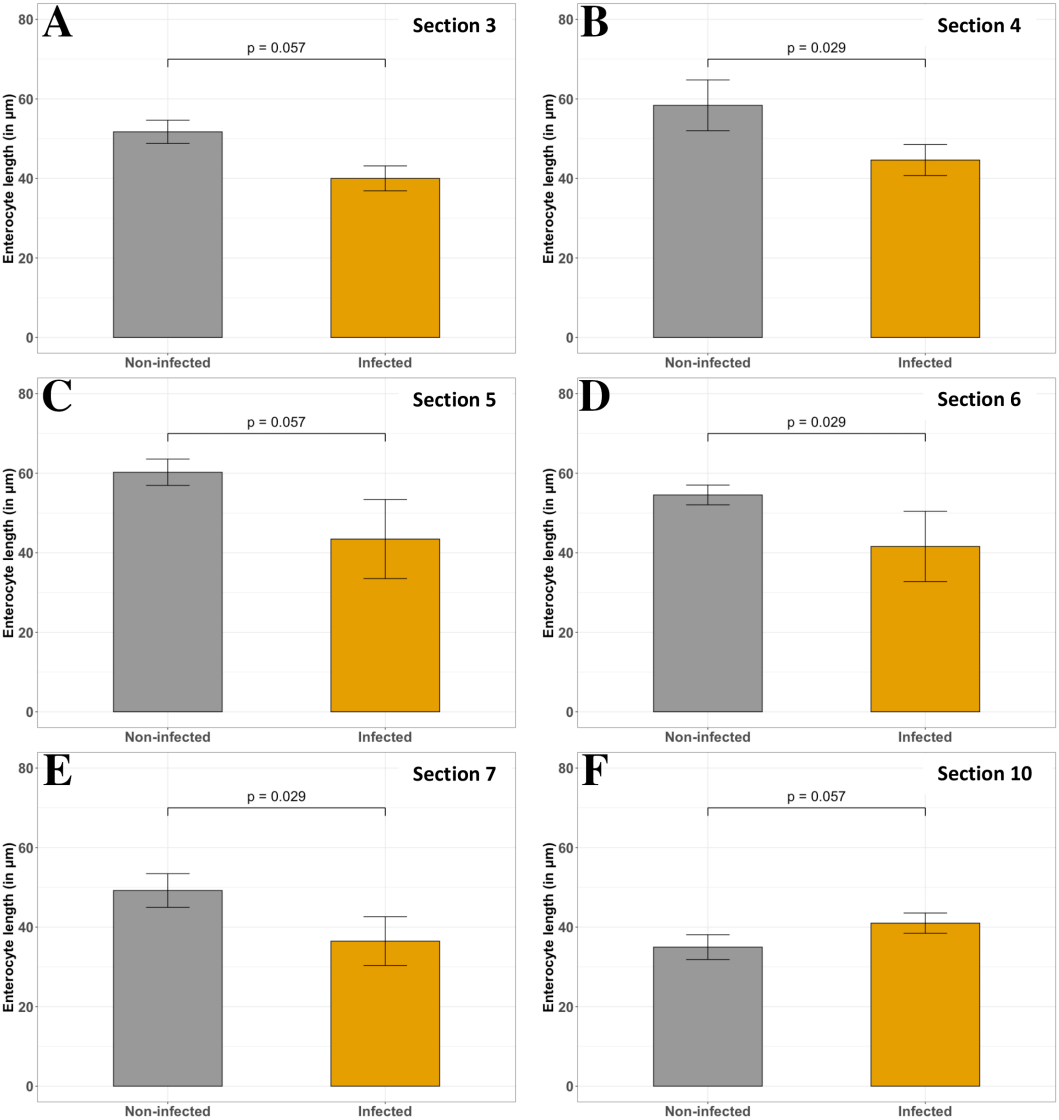
Comparison of non-infected and infected *P. orbicularis* enterocyte length (in µm) in several sections of GIT. (A–E) sections 3 to 7, respectively. (F) section 10. Grey and orange bar plots correspond to the average of enterocyte length measurements for non-infected and infected fish, respectively. Measurements have been assessed on slides stained with HES. Results of Wilcoxon test (*p*-value) is indicated for each comparison (*α* = 0.05, *n* = 4 per treatment).

*T. maritimum* infection impacted the quantity and quality of the mucous (Figs. 7, 8 and 9). In the duodenum and pyloric caeca sections (sections 3 to 5) in non-infected fish, mucus appeared acidic along the bottom of the folds and became progressively neutral towards the tip of the folds. The proportion of acidic mucous cells is significantly higher in infected fish in the proximal duodenum compared with non-infected individuals (Figs. 7C–7D and 8A, section 4, *p* < 0.05, Wilcoxon test). The same trend is observed for the distal duodenum but without any significant results (Figs. 7E–7F and Fig. 8B, section 5, *p* > 0.05, Wilcoxon test). No difference has been noted in the pyloric caeca (Fig. 7A–7B). Mucous cell density is relatively constant and varies from 0.06 to 0.07 mucous cells per µm in sections 3 to 5 for infected and non-infected fish, respectively (*p* > 0.05, Wilcoxon test). The PAS-AB staining showed unexpected results with differential marking of the enterocyte cytoplasm and the basal lamina. The marking being present and more intense in infected fish from the caeca to the ileum (Figs. 7B, 7D, 7F, 7H, 7J, 7L).

#### Impact on the epithelial thickness

Enterocyte length of the ileum and the colon is not affected by the infection. In the proximal duodenum and jejunum segments (sections 4, 6 and 7, Figs. 9B, 9D, 9E, respectively), a significant decrease is observed (24 to 26%, respectively) in infected fish (*p* < 0.05, Wilcoxon test). Even if the same trend appears in the pylorus and distal duodenum segments (sections 3 and 5, Figs. 9A, 9C), with a decrease of 23 and 28%, respectively, the statistical analyses are not significant (*p* = 0.057, Wilcoxon test). On the contrary, in the rectum (section 10, Fig. 9F), epithelial thickness tends to slightly increase (+15%) in infected fish compared with non-infected fish. Finally, the vacuoles are absent in the jejunum segment of infected fish compared to non-infected fish (Figs. 4DD and 7J, 7L, sections 7 and 8).

#### NKA signal in enterocytes

NKA labelling in the esophagus and stomach in non-infected and infected fish is weak (Fig. 5). In all the samples, a strong NKA marking is observed in the anterior portions of the intestine, including the caeca (sections 3 to 5). The NKA signal is slightly weaker in the rest of the intestine. However, compared to control animals, difference was observed in the jejunal section (sections 6 and 7) where NKA signal appeared higher in infected individuals (Data S2).

## Discussion

This study provides new insights in the description of the GIT in a disc-shaped fish, the orbicular batfish *P. orbicularis*. Literature about this species in particular, and for Ephippidae in general, is very scarce. Furthermore, the emerging aquaculture industry using *P. orbicularis* is threatened partly due to *T. maritimum*, the aetiological agent of the tenacibaculosis, causing severe mortality episodes (Reverter et al., 2016). Although the tenacibaculosis is fatal, the mechanisms leading to this lethality are still poorly understood and complicated by the presence of opportunistic bacteria such as *Vibrio* sp (Avendaño Herrera, Toranzo & Magariños, 2006). To better understand the impact of these infections on *P. orbicularis*, we studied the effect of a non-invasive *T. maritimum* challenge on *P. orbicularis* GIT. Fish GIT is an interface with the environment and is involved in several key functions (Wilson & Castro, 2010; Ray & Ringø, 2014). By choosing to study the GIT, we expect providing new elements allowing us to better understand the etiology of this disease. However, due to the shape of the GIT of our species, it appeared necessary to first characterize it to better understand its functionalities.

Among teleosts, GIT structure and functional characteristics vary considerably according to feeding habits, ontogenic stages, environment, phylogeny and also the shape of the body (Kapoor, Smit & Verighina, 1976; Ray & Ringø, 2014). The coelomic cavity of *P. orbicularis* is highly restricted due to the discoid shape of this species which probably explains the overlapped organization of the GIT. Among the large diversity of teleost, this type of GIT organization is not unusual (e.g., Chanet et al., 2012; Aguilar-Medrano, Kobelkowsky & Balart, 2015; Kobelkowsky & Rojas-Ruiz, 2017). The ocean sunfish (*Mola mola*), a disc-shaped and laterally flattened species, as *P. orbicularis*, has a deeply coiled GIT (Chanet et al., 2012). The dusky flounder (*Syacium papillosum*), another example among the flatfish, shows the same GIT organization, including the liver place and number of pyloric caeca as our species of interest (Kobelkowsky & Rojas-Ruiz, 2017). These convolutions are also observed among teleost with long intestines, e.g., Cyprinidae, probably to compensate poor development of the intestinal folds in these species (Wilson & Castro, 2010). The convoluted shape of the GIT may challenged the digestive process resulting in some issues for food to be transported throughout the tract. Strong peristaltic movements and therefore muscles might be involved. In spite of the convolutions observed, the RIL or IC is relatively low (1.47 ± 0.14), compared to other species (from 0.5 to 13.0 depending on the species) and is in the range of carnivorous species (Ray & Ringø, 2014; Kalhoro et al., 2019). The RIL or IC is used as a morphometric marker to classify fish according to their feeding habits, age or development (Kalhoro et al., 2018; Kalhoro et al., 2019). However, the link between intestinal morphology (e.g., length) and fish diet due to specific requirements for the digestion is still controversial (Kapoor, Smit & Verighina, 1976; Wilson & Castro, 2010; Dala-Corte, Becker & Melo, 2017; Kalhoro et al., 2018; Kalhoro et al., 2019). Indeed, several studies about intestine plasticity highlighted the drastic changes in GIT length especially following starvation (Kapoor, Smit & Verighina, 1976; Zaldúa & Naya, 2014; Ramírez, Davenport & Mojica, 2015). Moreover, multiple additional factors come into play, such as salinity of the environment, osmoregulatory activity, size of the coelomic cavity and genetic traits. Individual variations within the same species have also been identified (Kapoor, Smit & Verighina, 1976; Collar et al., 2009; German et al., 2010). As far as known, the feeding habits of *P. orbicularis* are considered as very plastic and depend on the ontogenetic stage and the environmental conditions (Barros et al., 2013). However, due to the scarce information we possess about our species, further studies on diet and different ontogenic stages are necessary to make a conclusion.

Although the orbicular batfish GIT is highly convoluted compared to other teleost species, its organization is similar (e.g., (Løkka et al., 2013; Alix et al., 2017; Kalhoro et al., 2019; Vidal et al., 2020)). It presents a foregut (esophagus and stomach), a midgut (intestine) and finally, a short hindgut (rectum), separated by several identified junctions and valve. The transition between the esophagus and the stomach even if not well defined, can be identified using the color of the organ, muscle layers and the presence or absence of mucous subepithelial glands (Hellberg & Bjerkas, 2000; Ray & Ringø, 2014; Vidal et al., 2020). The stomach displays a siphonal (J or U) shape which is the most common type among fish (e.g., Eastman & DeVries, 1997; Løkka et al., 2013; Kalhoro et al., 2018). *P. orbicularis* has pyloric caeca as approximately 60% of teleosts (e.g., Pedersen & Falk-Petersen, 1992; Løkka et al., 2013; Aguilar-Medrano, Kobelkowsky & Balart, 2015; Alix et al., 2017; Kalhoro et al., 2018; Kalhoro et al., 2019; Vidal et al., 2020; Verdile et al., 2020). These blind-ended ducts are probably involved in the surface area increase for absorption and digestion (Pedersen & Falk-Petersen, 1992; Ray & Ringø, 2014). Their histological structure resembles to the intestine and more specifically to its anterior part according to the mucosa organization and mucus composition. In that latter regard, our results are similar to previous studies describing acidic mucins with a majority of sialomucin for the goblet cells in the intestine although with smaller amounts of sulfomucin (e.g., Kalhoro et al., 2019; Vidal et al., 2020). However, some exception are noticed for the pyloric caeca and anterior intestine in *P. orbicularis* in which acidic mucus is present at the base of the folds and become progressively neutral at the top of the folds, with intermediate mixed mucus. Neutral mucus may be involved in the lubrication of the intestine or in the protection of the folds whereas acidic mucus could play a role in nutrient absorption or against bacterial infection (Shephard, 1994; Tibbetts, 1997). Interestingly, in the distal part of the mid-intestine (ileum), a dense pink staining of the epithelium and underlying connective tissue has been observed which could correspond to carbohydrates absorption. Although the ability to digest carbohydrates is different according to fish species, glucose affinity increases from the proximal to the distal part of the intestine (Krogdahl, Hemre & Mommsen, 2005). In terms of absorption, vacuoles have been observed at the top of the folds in the distal part of the jejunum which could probably correspond to lipid or protein intake. However, according to the literature the majority of protein intake (80%) and lipid absorption take place in the anterior intestine whereas macromolecules absorption main site is the posterior intestine for various fish species (Wilson & Castro, 2010; Ray & Ringø, 2014). Finally, the rectum is distinguished from the rest of the GIT by a thickening of both muscle layers (particularly the circular layer), a reduction of the lumen size and the fold height as described in most of the teleost (e.g., Hellberg & Bjerkas, 2000; Vidal et al., 2020). These elements together with the presence of goblet cells are most probably associated with the defecation in this species.

The morpho-anatomy of the GIT can differ depending on the stage of development, age or environmental conditions (e.g., Gisbert, Piedrahita & Conklin, 2004; Alix et al., 2017). *Platax orbicularis* individuals challenged with the tenacibaculosis present as well changes, mostly in the jejunum section of GIT. This section is preferentially affected possibly due to the reduced amount of lymphoid tissue in this area (Rombout et al., 2011). One of the notable impacts is the reduction of the muscular layer thickness in infected individuals. The plasticity of the muscle layer depending on the microbiota has already been described (reviewed in Scirocco et al., 2016) and the reduction of muscle thickness is due to a de-differentiation of smooth muscle cells and a change in the extracellular matrix. In the present study, it would therefore be necessary to make additional staining such as collagen, alpha-actin or smoothelin to identify the structures potentially affected (Bitar, Raghavan & Zakhem, 2014). The changes in muscle layers could lead to a problem in bowel movement. Indeed, given the particularly folded and compacted morphology of *P. orbicularis* GIT, transit disorders are certainly possible because of the infection. In the intestine, transit is normally facilitated by waves of neutral mucus secretion. However, excess of mucus could also limit the absorption capacity of enterocytes. As already mentioned, mucus has a key role in immunity as a selective protective film (Capaldo, Powell & Kalman, 2017). Acidic mucus characteristic of sulfomucines could have a bactericidal action (Croix et al., 2011; Bergstrom & Xia, 2013). The proper functioning of the intestine therefore requires a good balance in the quality and quantity of mucus secreted. PAS-AB pH 2.5 analysis revealed a variation in the nature of the mucus secreted and in the proportion of mucous cells. Mucous cells with only neutral secretions almost disappear in the anterior sections of the intestine (sections 4 and 5) of infected individuals. Therefore the bactericidal action of mucous cells may be preferred over transit assistance in the event of infection with *T. maritimum*.

To refine the present results, the characterization of mucus using a PAS-AB pH1 staining (Lev & Spicer, 1964) would allow us to confirm the ratio of sulfomucines to sialomucines and a marking with lectin (e.g., isolectin B4) could also be used (Wong, Uchida & Tsukada, 2020). Nevertheless, the characterization of the nature of mucins does not seem to be an interesting approach as the intestine is mainly composed of Mucin-2 (MUC2) protein (gel-forming mucin) secreted by the mucous goblet cells (Bergstrom & Xia, 2013). The nature of MUC-2 *O*-glycosylations in the proline, threonine, serine-riche or ‘PTS’ domains seem to be discriminating and may be modified (Bergstrom & Xia, 2013). Among the mucin-type *O*-glycan, the sulfation may have a protective role (Bergstrom & Xia, 2013). Although sulfation seems to play a role in the isolation of pathogenic bacteria by mucus thickening, the nature of the interaction between bacteria and mucus glycoproteins are still to be investigated (McGuckin et al., 2011). The thickness of the mucus layer was not measured in the present study due to a fixative method not adapted to this measurement. The acquisition of new samples using an appropriate cacodylate fixation or performing cryo-cuts would also allow us to have data on the actual thickness of the mucus (Ermund et al., 2013; Suvarna, Layton & Bancroft, 2018).

Our analyses of PAS-AB pH2.5 staining, although basic (Yamabayashi, 1987), allowed us to reveal unexpected results on carbohydrate absorption stained by PAS (McManus, 1948). Indeed, what could have been taken as coloring artefacts, without the use of a TMA, shows a clear difference in carbohydrate uptake between infected and non-infected individuals. Infected individuals may try absorbing carbohydrates all along the intestine (starting from the anterior part) while non-infected individuals limit this absorption to the mid-intestine. Moreover, even in the jejunum, this absorption appears to be more important in infected individuals.

This predominant absorption of carbohydrate in infected individuals may characterize a metabolic choice to enhance ATP synthesis via catabolism of glucose in epithelial cells or other cells if carried by capillaries (Carroll, 2007; Sala-Rabanal et al., 2018). In this case, it could also lead to the accumulation of glucose as glycogen in the liver or muscles. The very strong staining of the basal lamina is consistent with absorption by the capillaries in the underlying connective tissue. This hypothesis is reinforced by the apparent decrease in the NKA pump observed in the duodenum. However, normally these pumps allow glucose absorption by pumping the sodium co-transported with it via the sodium glucose cotransporter SGLT1 (Whittamore, 2012). SGLT1 gene being particularly preserved, it would be valuable to confirm the results with analyses of SGLT1 mRNA expression (Syakuri et al., 2019) or *in situ* hybridization (Li et al., 2018; Zhang et al., 2019).

The decrease of the presence of the NKA pump may be related to the osmoregulation. The hypo-osmoregulatory activity of seawater teleost is shared between several organs whose intestines have a decisive role in early developmental stages (Sucré et al., 2009). The NKA results obtained in the present study are questionable though. The images obtained by confocal microscopy do not allow us measuring the intensity of the NKA marking. Technical problems in the mounting of the slides and the software interface settings resulted in bleaching and a lack of uniformity of several sections that have been detected during the process of image analysis. Consequently, these results are only of interest as a starting point to explore and confirm. Marking or quantifying the other transporters involved in osmoregulation (e.g., Na^+^/K^+^ /2Cl^−^ co-tranporter1, CFTR, AQP1, Claudin-3,-15,-25 ) would allow us determining the potential impact of *T. maritimum* on osmoregulatory activity (Whittamore, 2012).

ATP is also involved in the polymerization of the cytoskeleton (polymerization of actin microfilaments) and the myosin proteins activity as in enterocyte polarity (Schneeberger et al., 2018) and thus in the contraction of enterocytes and microvilli. The enterocyte length and particularly the brush border is related to a more intense absorption activity but also to the fight against bacterial infections (Shifrin & Tyska, 2012). In addition, the presence of luminous vesicles rich in alkaline phosphatase membrane and trapped in mucus provide better protection against infections (Shifrin & Tyska, 2012). Our work shows a significant decrease in the enterocyte length and a decrease in the vacuoles, presumed to be endocytosis of proteins in infected individuals. These results seems to be to the detriment of nutrition. However, TEM study of alkaline phosphatase staining (Mölbert, Duspiva & von Deimling, 1960) will be necessary to identify this type of vesicles.

In addition to the impact of the infection, the dynamic of the intestinal epithelium can be affected by a change in the differentiation of its cells during the animal’s development (Bates et al., 2006). It is difficult to say whether the juvenile stage of our study is affected by this type of change. However, it would be interesting to expose younger stages to *T. maritimum* to investigate the potential changes in the nature of their epithelium and give them better defense mechanisms.

**Figure 10:**
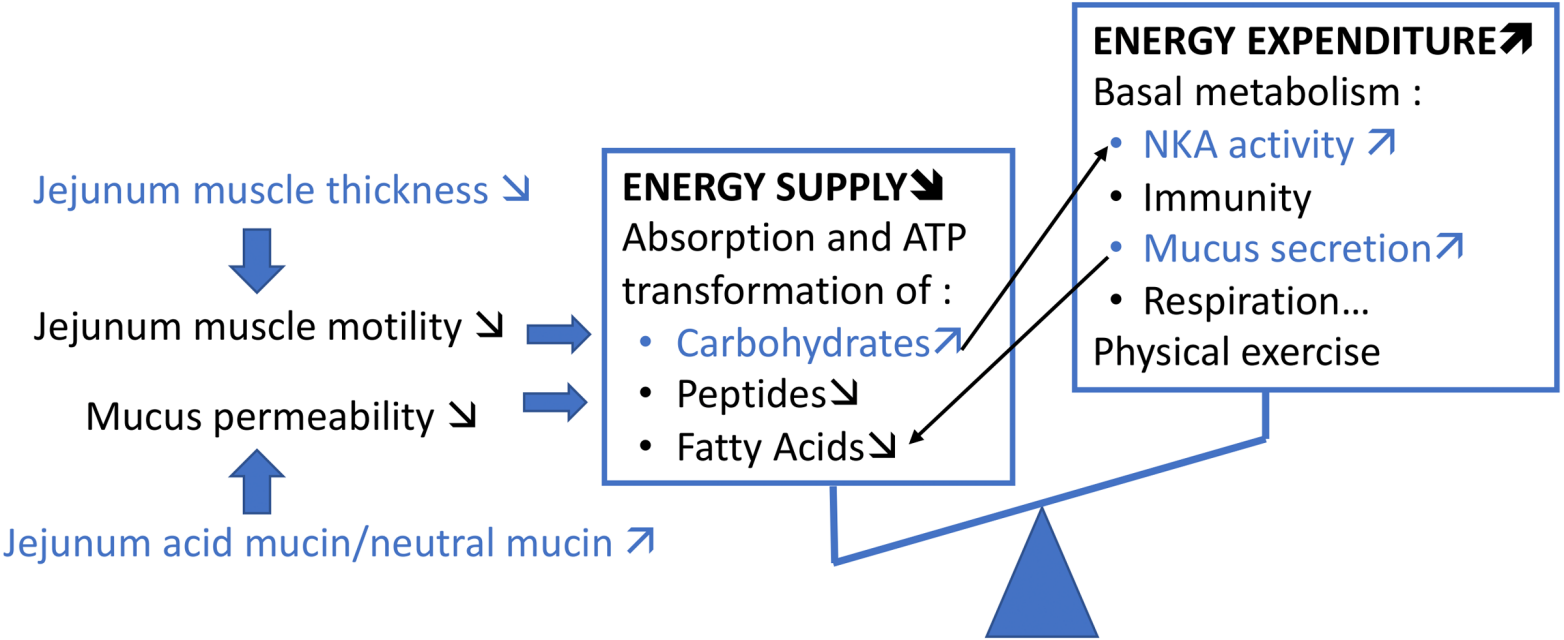
Schematic summary of our hypothesis on the potential impact of *T. maritimum* on the metabolic energy balance of *P. orbicularis*. Our results are represented in blue, the rest is only speculative and inspired from Pilch & Bergenhem (2006).

In the juvenile stage (56 dph), *T. maritimum* infection seems impacting the metabolic energy balance both by increasing energy expenditure and energy intake. Although some of our results remain to be confirmed (e.g., NKA increase), we can hypothesize that the energy imbalance, and a decrease in nutrition in particular, is the main cause of mortality (Fig. 10). The transit reduction probably due to thinner muscle layer could promote the absorption of carbohydrates. However, if the energy supplied by this way is rapidly expended by the NKA pumps and mucus secretion, it may not compensate the absorption decrease of peptides and fatty acids and the loss of energy caused. Indeed, the nature of the mucus seems to be related to the absorption of peptides with, among other things, a decline in absorption when the mucus is too viscous (Moran, 2016). The increase in mucus secretion would prevent the absorption of certain fatty acids and especially the short-chain fatty acids (Sakata, 2019). More in-depth studies of the markers of energy metabolism at the intestinal level are required to identify which imbalance in the trade-off between these functions causes the mortality of the infected individual.

## Conclusion

This work allows characterizing the different parts of the GIT of *P. orbicularis* juvenile and provides a key to identify them. The compacted GIT of the Ephippidae makes this tool essential for future studies to investigate the GIT sections and functions impacted in other environmental conditions. The juvenile stage considered is a milestone in terms of breeding because of the transition at this stage to lagoon cages and its first potential contact with *T. maritimum*. In our study, the infection impacted different structural levels of the intestine, e.g., acidification of the mucus, carbohydrate absorption increase and limited protein absorption. The most affected section, the jejunum, presented a significant decrease in the muscle layer thickness, enterocyte length and a high carbohydrate absorption. Finally, at the distal level, osmoregulatory activity seems to be favored by a stronger presence of NKA in the infected fish. Although several indicators are necessary to investigate to confirm this hypothesis, our first results point in the direction of an imbalance in the nutritional function as if the intestine reacted to *T. maritimum* by giving priority to protection. The decrease of nutrient intake (lipids and protein) and the absorption of glucose to supply energy to these protection mechanisms must be confirmed. However, if our hypothesis is confirmed, finding a nutritional supplementation pathway might be the solution to minimize the impact of the tenacibaculosis and to help the production of the species impacted by this disease.

##  Supplemental Information

10.7717/peerj.9966/supp-1 Supplemental Information 1 Illustration of whitish patches caused by *T. maritimum* on the skin of *P. orbicularis* (A) 56 days post-hatching (dph) *P. orbicularis*, picture from D. Saulnier, (B and C) 56 dph *P. orbicularis*, micrograph from A. Bantz using a stereoscopic microscope at magnification ×10 and ×35 respectively.

10.7717/peerj.9966/supp-2 Supplemental Information 2 Comparison of Na^+^/K^+^ ATPase labeling in different sections of *P. orbicularis* GIT in non-infected and infected fish using different settings for image correction On the Leica Microsystems TCS-SPE confocal microscope, the 488nm He/Ne laser line was at 14%, the PMT had a gain of 872.0 HV with 0% offset and the absorbed signal was between wavelengths 509.9 and 532.6 nm. The objective used on the DM5500 microscope was the PL FLUOSTAR 25.0 × 0.75 IMM. The pinhole was 94.4 µm and 1 airy. The obtained image is zoomed 1.5 times with a 1024x1024 pixel format and a resolution of 8bits. In spite of a limitation of the background noise (offset = 0%) the images obtained remain with unspecific signal of cytoplasm. In order to display only the specific signal, the light and the contrast have been adjusted to 20% and 40% respectively. No signal can be seen in the stomach and esophagus using these settings. However, if the light is adjusted to 50% and the contrast to 20%, the staining in the stomach and esophagus can be visualized but the anterior portions are overexposed and the differences between infected and unexposed individuals are difficult to distinguish. Scale bars = 25µm.

10.7717/peerj.9966/supp-3 Supplemental Information 3 Measurements of the different muscles layers in the sections of the digestive tract in non-infected and infected *Platax orbicularis* Longitudinal (ML), circular (MC) and total (MT) muscle layer thickness (in µm) have been assessed on slides stained with HES from section 4 to 10. Columns 3 to 5 correspond to the data used for the statistical analysis and presented in the Figs. 6C, 6D and 6E for the section 6 only.

10.7717/peerj.9966/supp-4 Supplemental Information 4 Measurements of the proportion of acidic mucocytes in different sections (3, 4 and 5) of the digestive tract in non-infected and infected *Platax orbicularis* Proportions of acidic mucocytes (in %) were measured on different histological slides stained with the PAS A.B pH 2.5. Column 4 corresponds to the data used for the statistical analysis and presented in the Figs. 8A and 8B.

10.7717/peerj.9966/supp-5 Supplemental Information 5 Measurements of the enterocyte length in different sections of the digestive tract in non-infected and infected *Platax orbicularis* Enterocyte length (in µm) have been assessed on slides stained with HES. Column 4 corresponds to the data used for the statistical analysis and presented in the Fig. 9.

## Additional Information and Declarations

### Data Availability

The following information was supplied regarding data availability: The raw measurements are available as Files S3–S5.
