# Supplementary figures and images for: Description of the unusual digestive tract of Platax orbicularis and the potential impact of Tenacibaculum maritimum infection
Source: PeerJ. 2020 Sep 24;8:e9966. doi: 10.7717/peerj.9966 (PMC7520087; doi:10.7717/peerj.9966)

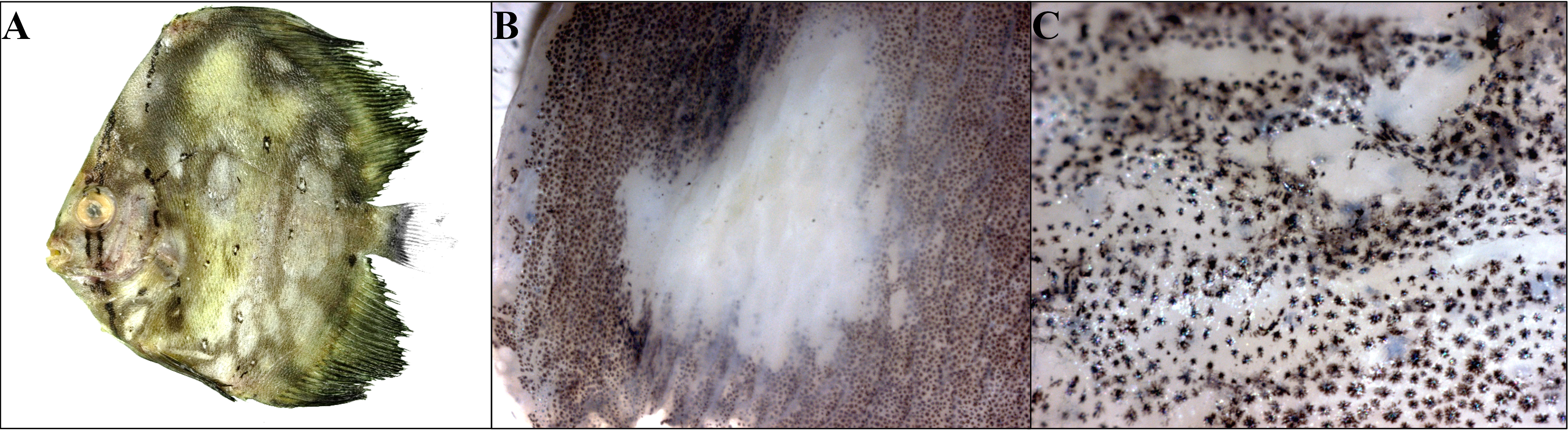

Supplement: Supplemental Information 1 — (A) 56 days post-hatching (dph) P. orbicularis, picture from D. Saulnier, (B and C) 56 dph P. orbicularis, micrograph from A. Bantz using a stereoscopic microscope at magnification ×10 and ×35 respectively. [file peerj-08-9966-s001.png]

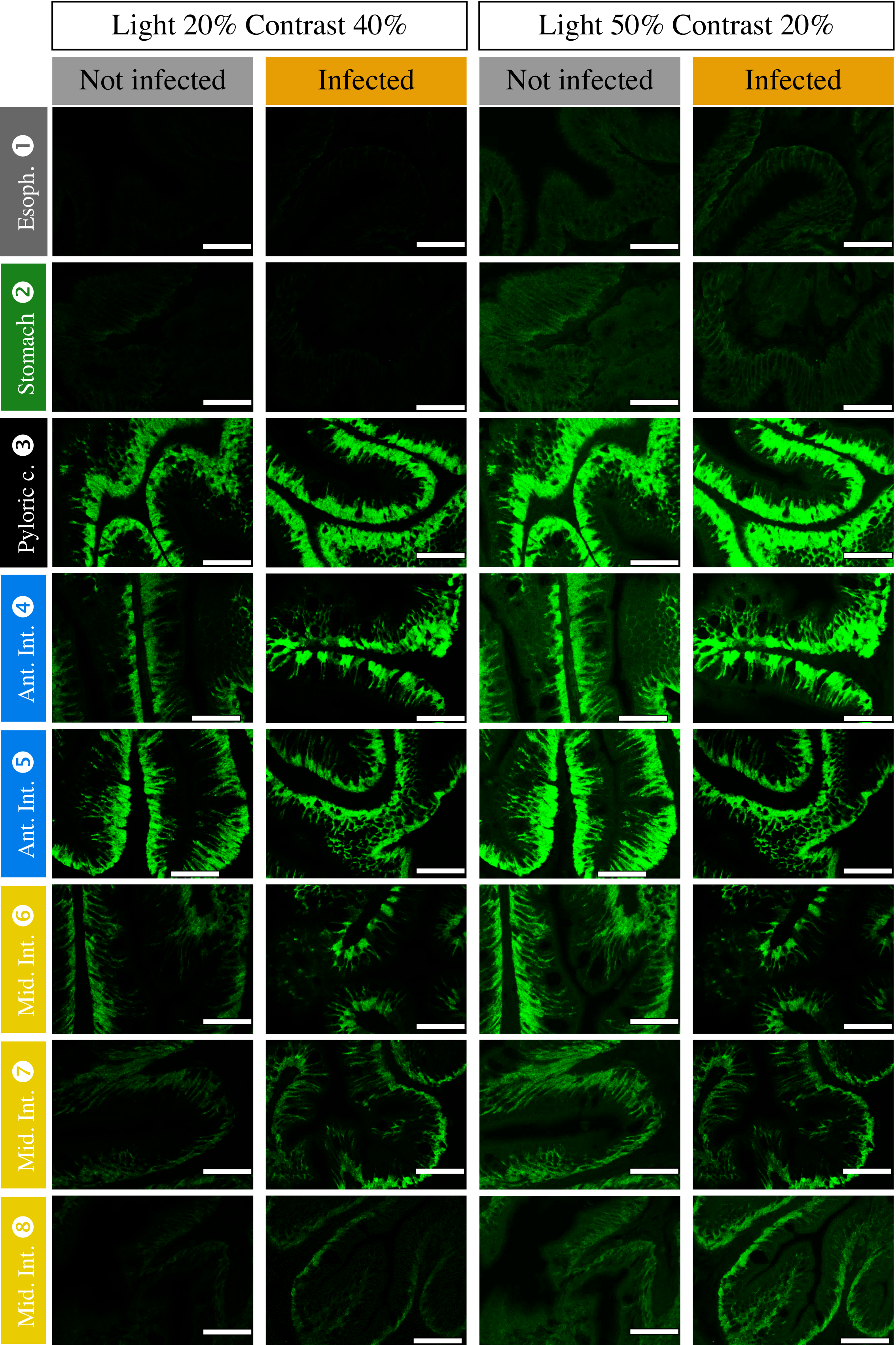

Supplement: Supplemental Information 2 — On the Leica Microsystems TCS-SPE confocal microscope, the 488nm He/Ne laser line was at 14%, the PMT had a gain of 872.0 HV with 0% offset and the absorbed signal was between wavelengths 509.9 and 532.6 nm. The objective used on the DM5500 microscope was the PL FLUOSTAR 25.0 × 0.75 IMM. The pinhole was 94.4 µm and 1 airy. The obtained image is zoomed 1.5 times with a 1024x1024 pixel format and a resolution of 8bits. In spite of a limitation of the background noise (offset = 0%) the images obtained remain with unspecific signal of cytoplasm. In order to display only the specific signal, the light and the contrast have been adjusted to 20% and 40% respectively. No signal can be seen in the stomach and esophagus using these settings. However, if the light is adjusted to 50% and the contrast to 20%, the staining in the stomach and esophagus can be visualized but the anterior portions are overexposed and the differences between infected and unexposed individuals are difficult to distinguish. Scale bars = 25µm. [file peerj-08-9966-s002.png]
